# The short-term impact of probiotic consumption on the oral cavity microbiome

**DOI:** 10.1038/s41598-018-28491-x

**Published:** 2018-07-11

**Authors:** Erik Dassi, Pamela Ferretti, Giuseppina Covello, HTM-CMB-2015, Alessandra Speccher, Alice Migazzi, Bartolomeo Bosco, Bodike Rajashekar, Calogero Zarbo, Claudio Ballabio, Daniele Rossetto, Eleonora Maino, Eloina Corradi, Federica Costa, Francesca Precazzini, Harun Or Rashid, Manuel Nicolussi, Mattia Bolzan, Michele Demozzi, Michele Olivieri, Nicole Zordan, Renato Pedron, Serena Manara, Setareh Rezvan, Sindhu Narasimha Naik, Solmaz Khaghani, Stefania Masella, Thomas Perli, Virginia Pierini, Roberto Bertorelli, Michela A. Denti, Veronica De Sanctis, Adrian Tett, Nicola Segata

**Affiliations:** 1 Centre for Integrative Biology, University of Trento, Trento, Italy; 2 NGS Facility, Laboratory of Biomolecular Sequence and Structure Analysis for Health, Centre for Integrative Biology, University of Trento, Trento, Italy; 3 High Throughput Methodologies Class 2014/2015, Master in Cellular and Molecular Biotechnologies, University of Trento, Trento, Italy

## Abstract

The dysbiosis of the oral microbiome is associated with both localized and systemic diseases. Modulating the resident microbial communities by the dietary consumption of probiotics has become an appealing means to promote host health by either restoring host-microbe balance or preventing dysbiosis. Most probiotics strategies target the intestinal microbiome, but little is known about their impact on the oral microbiome. We analyzed here the saliva microbiome from 21 volunteers, longitudinally collected before, during, and after consumption of a commercial probiotic and a standard yoghurt using 16S amplicon sequencing. The alpha diversity of the saliva microbiome had a statistically significant increase (P-value = 0.0011) in one of the groups that consumed the probiotic. The overall structure of the microbiome was however not significantly impacted by the probiotic, although oligotyping analysis revealed that both *Streptococci* and *Lactobacilli* present in the probiotic product persisted in the saliva microbiome. In contrast, non-probiotic yoghurt consumption had a lesser impact on the overall diversity and *Lactobacillus* and *Streptococcus* persistence. Our results suggest that consumption of commercial probiotics in healthy subjects increase the overall diversity of the oral cavity microbiome in the short term, but such dietary interventions are not able to substantially modify the structure of the microbiome.

## Introduction

The role of the human microbiome in human health has been investigated extensively, highlighting its importance in immune system regulation^1,2^, nutrient absorption^3^, and weight balance^4^. While the majority of these studies have focused on the gut microbiome, the oral microbiome has also received attention^5,6^, particularly in association with the initiation and development of common oral diseases such as dental caries^7–9^.

The potential to modify the microbiome by introducing live microbial strains that are presumed to be beneficial to health, so called probiotics, is an active area of research. In this context, most studies have investigated the impact of probiotic bacterial species on the gut microbiome. Such bacteria usually belong to the *Bifidobacterium*, *Lactobacillus* and *Streptococcus* genera^10,11^, and their potential beneficial effects have been investigated both in mouse models^12–14^ and human trials^15–17^. While several products are commercially available for the modulation of the human gut microbiome^18,19^, only a few have been specifically proposed for the oral cavity^20^.

We previously described the short-term changes induced by a commercial probiotic product in the taxonomic composition of the saliva microbiome^21^. This study suggested that the overall diversity of the microbiome increased with the consumption of a probiotic product, but because of the limited sample size and the cross-sectional rather than longitudinal study design, additional evidence is required. In this study, we further investigate the impact of a probiotic and standard yoghurt intake in terms of their effect on the composition and structure of the oral microbiome and evaluate the persistence of the probiotic strains.

## Results

### The saliva microbiome after consumption of a probiotic product

**Figure 1:**
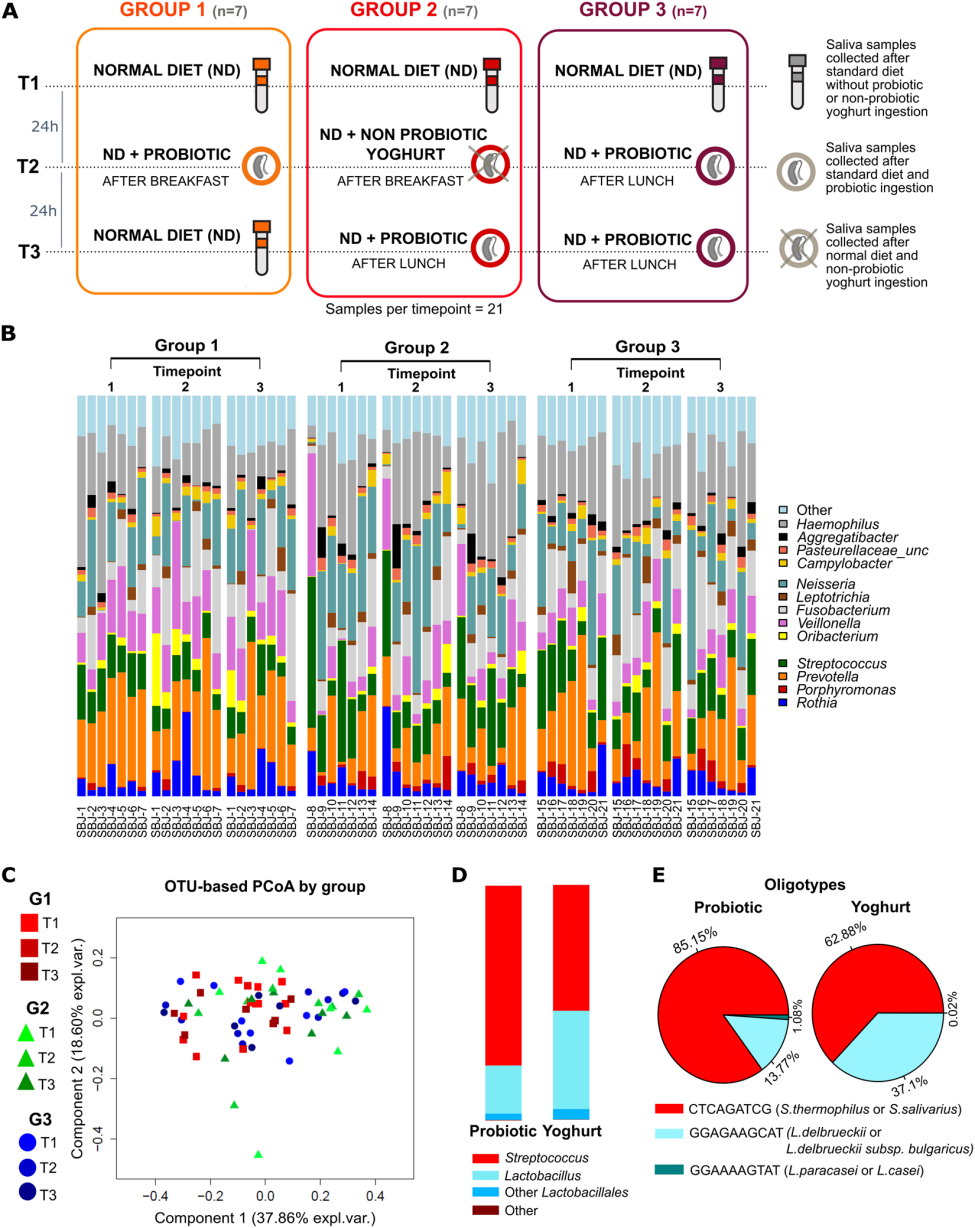
Study design, composition and diversity of the saliva microbiome. (**A**) Study design comprising three groups of seven healthy volunteers each. The baseline is defined at T1, without any probiotic or non-probiotic yoghurt ingestion. Group 1 was designed to evaluate the influence of a single probiotic intake, while Group 2 was designed to assess the differences between probiotic and non-probiotic yoghurt consumption. The last group was used to investigate a repeated probiotic intake (after lunch at both T2 and T3). All samples were collected after lunch, while yoghurt or probiotic ingestion occurred at breakfast (~5 hours before sample collection) or at lunch (~1 hour before sample collection) as reported in the figure. (**B**) Taxonomic profiling at genus level for all groups and timepoints. (**C**) Principal Coordinates Analysis (PCoA) plot of OTU-based beta diversity (weighted Unifrac) with samples colored by group. (**D**) Quantitative taxonomic composition of the probiotic fermented milk drink (“Probiotic”) and the non-probiotic commercial yoghurt (“Yoghurt”) as derived by OTU analysis and (**E**) by oligotyping analysis.

In this study we collected longitudinal saliva samples from 21 healthy volunteers, divided into three groups of seven volunteers (Fig. 1A). Each of these three group was asked to follow a probiotic-free diet for at least two weeks, after which the first set of saliva samples were collected to define the baseline microbiome configuration (T1). The following day (T2), the first and the third group of volunteers consumed the probiotic product (at breakfast and lunch respectively) consisting of a commercial fermented milk drink (“Coop Probiotico Bianco” [COOP Italia s.c. Bologna (IT)], 100 gr) containing *S. thermophilus, L. delbrueckii* subsp. *Bulgaricus* and *L. paracasei* strains, while the second group consumed the same amount of a non-probiotic yoghurt (“Mila Bianco” [Mila latte montagna, Alto Adige (IT)], 100 gr). On the last day of sampling (T3), the first group reverted back to the normal diet while the second and the third groups ate the probiotic product.

The saliva microbiome was investigated by 16S rRNA gene sequencing producing a total of 4.6 million reads (39,3 k ± 14,2 k reads per sample, Supplementary Table 2). We also sequenced the probiotic and non-probiotic products to assess their composition (56,3 k and 12,0 k reads respectively). By applying the QIIME pipeline^22^ to the dataset (see Methods), we generated a total of 4503 operational taxonomic units (OTUs) clustered at 97% identity with an open-reference OTU picking strategy.

The three most abundant phyla in the saliva samples were Proteobacteria, Firmicutes and Bacteroidetes, with average relative abundances of 36.4%, 28.7%, and 18%, respectively. Fusobacteria (10.5%) and Actinobacteria (5.5%) were less abundant, but still present in all samples (minimum abundance of 0.2% and 0.9% respectively) (Supplementary Figure 1A). At the genus level, *Prevotella*, *Neisseria*, *Haemophilus*, and *Streptococcus* were the dominant genera (Fig. 1B), which is consistent with previous studies of the healthy oral microbiome^6,23^. The three sets of biological replicates we performed showed a very low technical variability (Supplementary Figure 1B).

The complexity of the saliva microbial communities in subjects who ate the probiotic drink tended to be higher than for those who ate the standard yoghurt (Supplementary Figure 2A), confirming our previous finding^21^ although the result was not statistically significant (P-value 0.081). The samples associated with repeated probiotic intake were characterized by a higher alpha diversity than standard diet and single probiotic intake ones (Supplementary Figure 2C) but, again, this was not supported by statistical significance.

Samples did not show a strong grouping by probiotic intake when displaying their inter-sample beta diversity with ordination plots (Fig. 1C), which was expected based on our previous observations^21^. Instead samples tended to cluster by subject, according to both standard OTU analysis and subspecies level oligotyping analysis^24^ (Supplementary Figure 3) of the four most abundant genera (*Streptococcus*, *Haemophilus*, *Prevotella*, and *Neisseria*), confirming the presence of a personal microbial signature in the saliva^25,26^ (Supplementary Figures 4 and 5). We also profiled both the probiotic fermented milk drink and the non-probiotic yoghurt (Fig. 1D) finding that, in both communities, *Streptococcus* and *Lactobacillus* were the most dominant genera as these encompass the bacteria responsible for fermentation of the products. In the probiotic product, *Streptococcus* was enriched (76%) compared to the non-probiotic (53%), while *Lactobacillus* was characterized by an opposite trend (20% in probiotic, 41% in non-probiotic yoghurt). This is compatible with the known composition of the probiotic, enriched in *S. thermophilus*, *L. delbrueckii* subsp*. bulgaricus* and *L. paracasei* strains. Both the probiotic and non-probiotic product shared the most abundant *Streptococcus* oligotype and the four most abundant *Lactobacillus* oligotypes (Fig. 1E).

### Evidence of increased complexity of the oral microbiome after short-term probiotic consumption

**Figure 2:**
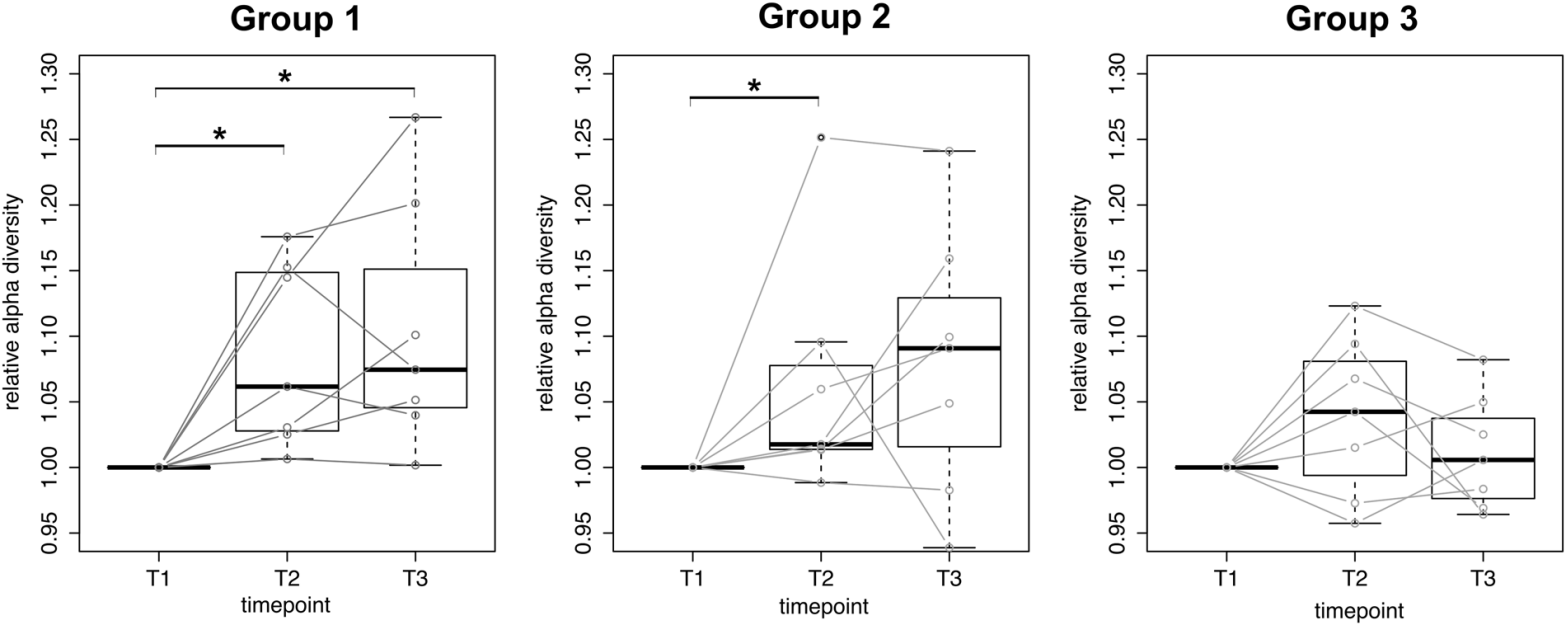
Longitudinal alpha diversity (PD-WT metric) across groups normalized to the baseline (T1). (* for P-values <0.05).

The longitudinal analysis of the alpha diversity in the three groups (Fig. 2 and Supplementary Figure 6) revealed that probiotic intake is significantly associated with an increase in the microbial community diversity for Group 1 (P-value of 0.0011 between T1 and T2). The non-probiotic yoghurt consumption group also showed an increased alpha diversity but to a lesser extent than its probiotic counterpart (P-value of 0.0204 between T1 and T2 in Group 2). Group 3 also consumed the probiotic, but the observed increase in average alpha diversity was not significant. Probiotic consumption is thus associated with a higher, albeit limited, enrichment in the microbial complexity of the saliva, compared to the non-probiotic yoghurt and standard diet.

We further investigated the differences in microbiome composition across time-points and study groups. Ordination analysis showed no apparent clustering by group, suggesting that the overall structure of the saliva microbiome is not affected by short-term probiotic intake (Fig. 1C). Indeed, no statistically relevant difference was found in beta diversity analysis across timepoints and groups (Supplementary Figure 7). Moreover, no significant variations were observed when comparing intra- and inter-samples distances at different timepoints (Supplementary Figure 8). Although the probiotic consumption does not change the microbiome composition to a large extent, the large overall variability could potentially hide the small-scale changes driven by probiotic intake but biomarkers detection analysis with multiple hypothesis testing correction^27^ resulted in no statistically significant differentially abundant features.

Overall, despite an increase in microbiome complexity after probiotic intake, no strong variation in the taxonomic structure of the saliva microbiome was observed.

### Persistence of the probiotic strains in the saliva microbiome

**Figure 3:**
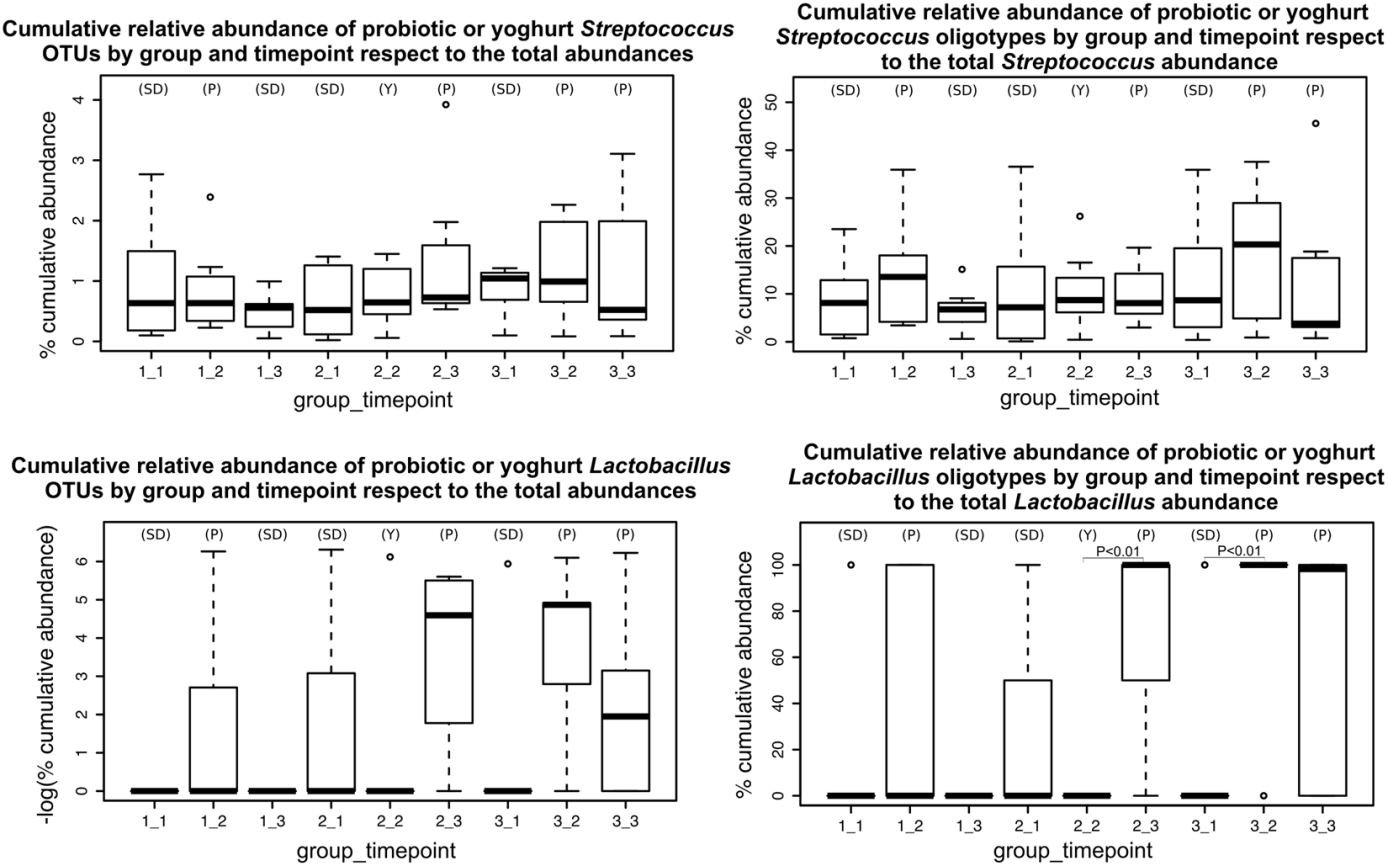
Relative abundances of Streptococcus and Lactobacillus OTUs and oligotypes across timepoints and study groups*. (SD) shows the standard diet, (Y) the non-probiotic yoghurt and (P) the probiotic intake*.

We then investigated whether bacteria present in the probiotic product (*S. thermophilus*, *L. delbrueckii* subsp*. bulgaricus* and *L. paracasei*) were detectable in the saliva samples to evaluate the presence and persistence of the probiotic strains in the saliva. As a genus, *Streptococcus* was present in all subjects, and even OTU analysis cannot reach the resolution needed to track the probiotic strain. Nevertheless, finer resolution oligotyping showed a higher abundance of the probiotic *Streptococcus* oligotypes in subjects who ate the probiotic with respect to non-probiotic oligotypes of the same genus (37.1% average increase after probiotic consumption in Group 1 and 28.7% average increase in Group 3, P-values: 0.37 and 0.51, respectively) (Fig. 3). When the subjects reverted to the standard diet also the probiotic-specific *Streptococcus* oligotypes returned to a condition similar to the baseline, confirming the short time persistence of oral probiotic organisms (P-value: 0.17, 51.6% average decrease in the only group with standard diet following the probiotic consumption timepoint, Group 1). The same oligotyping analysis performed for probiotic *Lactobacillus* highlighted a stronger effect compared to *Streptococcus*, probably because the former is more likely to be confounded with other *Streptococci* commonly present in the human saliva. The probiotic *Lactobacillus* OTUs and oligotypes were indeed found enriched (P-values OTUs for the three study groups: 0.21, 0.12 and 0.22; P-values oligotypes: 0.27, 0.008 and 0.004) only in groups which consumed the fermented milk product (*Lactobacillus* oligotypes relative abundances increased on average 66.7% and 83.3% after probiotic consumption at breakfast in Group 1 and at lunch in Group 3, respectively), with the only exception of the first time-point of the second group (associated with standard diet), in which the *Lactobacillus* oligotypes were present at relatively high abundance (28.6%). In contrast, no significant enrichment was found associated with the non-probiotic yoghurt consumed at breakfast (Fig. 3). In general, the probiotic-associated *Lactobacillus* OTUs and oligotypes showed a longer term persistence in the oral microbiome than the *Lactobacillus* OTUs and oligotypes associated with the non-probiotic yoghurt intake.

## Discussion

In this work we extended our previous analysis^21^ to verify the hypothesis that the intake of commercially available probiotic products has a directly effect on the diversity and composition of the saliva microbiome, at least at short timescales. Our longitudinal study design showed that the diversity of the saliva microbiome is increased after consumption of the probiotic product. The overall taxonomic and abundance distribution of bacterial genera is however minimally influenced by probiotic intake even though we verified that the bacteria present in the probiotic product persist to a certain extent in the saliva microbiome. In particular, the impact of probiotic *Streptococci* is limited in that it only slightly modifies the overall abundance of this already resident genus. Stronger support for short-term persistence was found for *Lactobacillus*, probably because this organism is not common in the oral microbiome. Altogether, our study confirms that the oral microbiome can potentially be modulated by commercial probiotic products, but that this modulation is limited in both the breadth of the change and in its temporal persistence. Additional studies are required to verify whether continued (e.g. daily) intake of probiotics for longer periods can have a larger and more long-lasting impact on the structure of the oral microbial communities.

## Methods

### Cohort and Study design

Saliva samples were collected from 21 age-matched healthy volunteers (10 males, 11 females, avg. age 23 s.d. 1,23) that were sampled at three different time-points, following the protocol in Fig. 1A and as described below. We collected 21 samples for each time-point and, for the first time-point, three samples were collected in duplicate as controls for a total of 66 samples. All participants gave their informed consent, and this study was approved by the ethical committee of the University of Trento (Reference number 2015-014); all experiments were performed in accordance with the relevant guidelines and regulations for human subjects research. Each volunteer completed a questionnaire about oral care habits (including frequency of teeth brushing, use of dental floss or mouthwash). Current smokers and individuals using antibiotic treatments in the last six months were excluded from the study. Volunteers were instructed to self-collect the saliva samples and were asked not to brush their teeth, use dental floss or mouthwash before sample collection. They were also requested to avoid intimate kissing. Self-collection was performed under supervision and directly in the laboratory. Volunteers were randomly assigned into three groups of seven subjects (Fig. 1A). For all the three groups, no administration of probiotic or non-probiotic yoghurt was perfomed for the first time-point in order to define the baseline. The second collection after the probiotic administration for the first and the third group and the non-probiotic yoghurt for the second group. At the third sampling, the first group returned to the standard diet, the second switched to the probiotic and the third group repeated the probiotic intake. The probiotic was a commercial fermented milk drink (“Coop Probiotico Bianco” [COOP Italia s.c. Bologna (IT)], 100 g) enriched in *S. thermophilus*, *L. delbrueckii* subsp. *bulgaricus*, *L. paracasei* strains and supplemented with vitamin B6 and vitamin D, conserved at +4 °C until consumption. The same amount of non-probiotic commercial yoghurt (“Mila Bianco” [Mila latte montagna, Alto Adige (IT)], 100 g) was administered as a control.

### Sample collection and DNA extraction

Samples, including technical replicates, were collected through a passive drooling procedure in sterile 50 ml screw top tubes (approximately 3 ml of saliva per sample). About 30 minutes before collection, all volunteers performed a mouth rinse with drinking water. Samples were vortexed before 2 ml of each was centrifuged at 10,000 × g for 10 minutes to collected the bacterial pellets. Total DNA was extracted using the PowerSoil DNA Isolation Kit (MoBio), according to manufacturer’s instructions. The same extraction procedure was also performed on 2 ml of the probiotic drink, the non-probiotic yoghurt and pure water as a negative control. DNA was eluted in 100 μl molecular-grade water and the DNA purity, yield, and concentration was assessed with the NanoDrop ND-2000 spectrophotometer (Thermo Scientific, Waltham, MA, USA). DNA was stored at −20 °C until used.

### 16S rRNA hypervariable region V4 amplification library preparation and sequencing

The V4 hypervariability region of 16 rRNA gene was amplified using Illumina adapted primer 515F (GTGYCAGCMGCCGCGGTAA), and Illumina adapted barcoded 806R primers (GGACTACNVGGGTWTCTAAT) as described previously^28,29^. The complete list of sample-specific barcodes is included as supplementary material (Supplementary Table 1). Each sample was PCR amplified in duplicate 25 µl reactions each comprising: 10 µl 5 PRIME HotMasterMix (Invitrogen; Life Technologies Europe BV, Monza, Italy) 0.5 µl each of forward and reverse primers (final concentration of 200 nM), 1 µl template DNA (10 ng/µl) and PCR grade water up to the final volume of 25 µl. The thermal cycling conditions were: 94 °C 3 minutes initial denaturation, 35 cycles of 94 °C (45 s), 50 °C (60 s), 72 °C (90 s) followed by a final extension of 10 minutes at 72 °C.

The duplicate amplicon libraries were pooled, visualized and extracted from a 1.5% agarose gel using the Wizard SV Gen and PCR Clean-Up System (Promega Italia s.r.l., Milano, Italy), as described by the manufacturer. The libraries were further cleaned using Agencourt AMPure Xp purification (Beckman Coulter s.r.l, Milan, Italy), following manufacturer’s instructions, and quantified using the Qubit Fluorometric Quantitation system (ThermoFisher Scientific-Invitrogen; Life Technolo-gies Europe BV, Monza, Italy). Finally, equal molarities of amplicons were pooled and sequencing was performed with Illumina MiSeq Reagent Kit v2 (300 cycles) as described in^29^.

### Data analysis

The Illumina MiSeq sequencing run produced 4.6 million paired-end reads after merging with fastq-join^30^ using default parameters. Joined reads had an average length of 250 bp. We obtained on average 39.3 thousands reads for each sample. Average phred quality score per read was 37. QIIME^22^ was used to demultiplex and trim low quality reads, reducing the initial reads to 3.38 million (26.5% reduction on average). Trimming was performed with 25 as minimum acceptable quality and 75% as minimum acceptable read length after trimming. FastQC^31^ was used to analyze overall quality of the raw data. The median sequence length of joined reads after pre-processing was 253 bp. Operational Taxonomic Units (OTU) were built using 5 as minimum OTU cardinality using UCLUST^32^. The few OTUs present also in the negative controls were discarded. RDP^33^ was then used to perform the taxonomic profiling, using Greengenes 13.8 dataset as reference sequences^34^. QIIME was also used to measure alpha diversity and beta diversity. All ordination plots are based on PD-whole tree metric, if not otherwise specified. Biomarker detection was performed using LEfSe^27^. Oligotyping^24^ analysis was performed by extracting reads assigned by QIIME to each genus (*Streptococcus*, *Lactobacillus*, *Haemophilus*, *Neisseria*, *Prevotella*) and using 10 entropy components for *Streptococcus* and *Lactobacillus*, 5 for *Neisseria*, 9 for *Haemophilus* and 15 for *Prevotella*; minimum substantive abundance filter threshold was set to 50 reads in all cases.

### Ethics approval and consent to participate

This study was approved by the ethical committee of the University of Trento (Reference number: 2015-014) and all participants gave their signed informed consent prior to enrollment.

### Consent for publication

Not applicable because no individuals person’s data are reported in any form.

### Data availability

Raw sequences were deposited in the NCBI Short Read Archive (http://www.ncbi.nlm.nih.gov/sra) and are available with accession numbers SRR2093962 to SRR2094047.

## Electronic supplementary material

Supplementary Information

Supplementary Table 1

Supplementary Table 2
