# Supplementary material for: The short-term impact of probiotic consumption on the oral cavity microbiome
Source: Sci Rep. 2018 Jul 11;8:10476. doi: 10.1038/s41598-018-28491-x (PMC6041349; doi:10.1038/s41598-018-28491-x)
Supplement: Supplementary file 1 — Supplementary Information [file 41598_2018_28491_MOESM1_ESM.pdf]

The short-term impact of probiotic consumption on the oral cavity microbiome

Erik Dassi^1, Pamela Ferretti^1, Giuseppina Covello[1], HTM-CMB-2015, Roberto Bertorelli[2], Michela A. Denti[1], Veronica De Sanctis[2], Adrian Tett[1], Nicola Segata*[,1]

Supplementary Figures

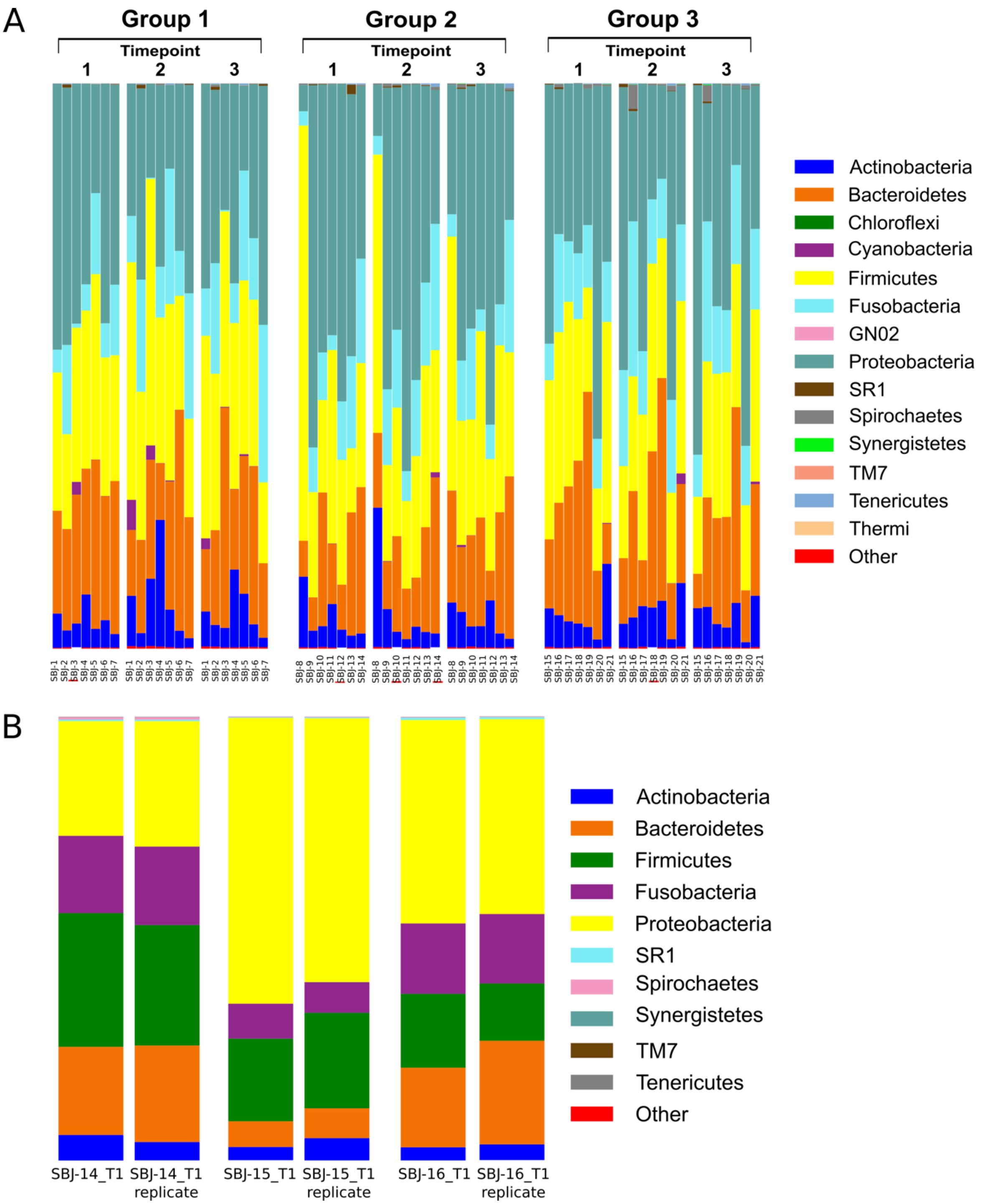

Supplementary Figure 1. (**A**) Taxonomic profiling of all samples at the phylum level. (**B**) Comparison of replicates taxonomic composition at the phyum level.

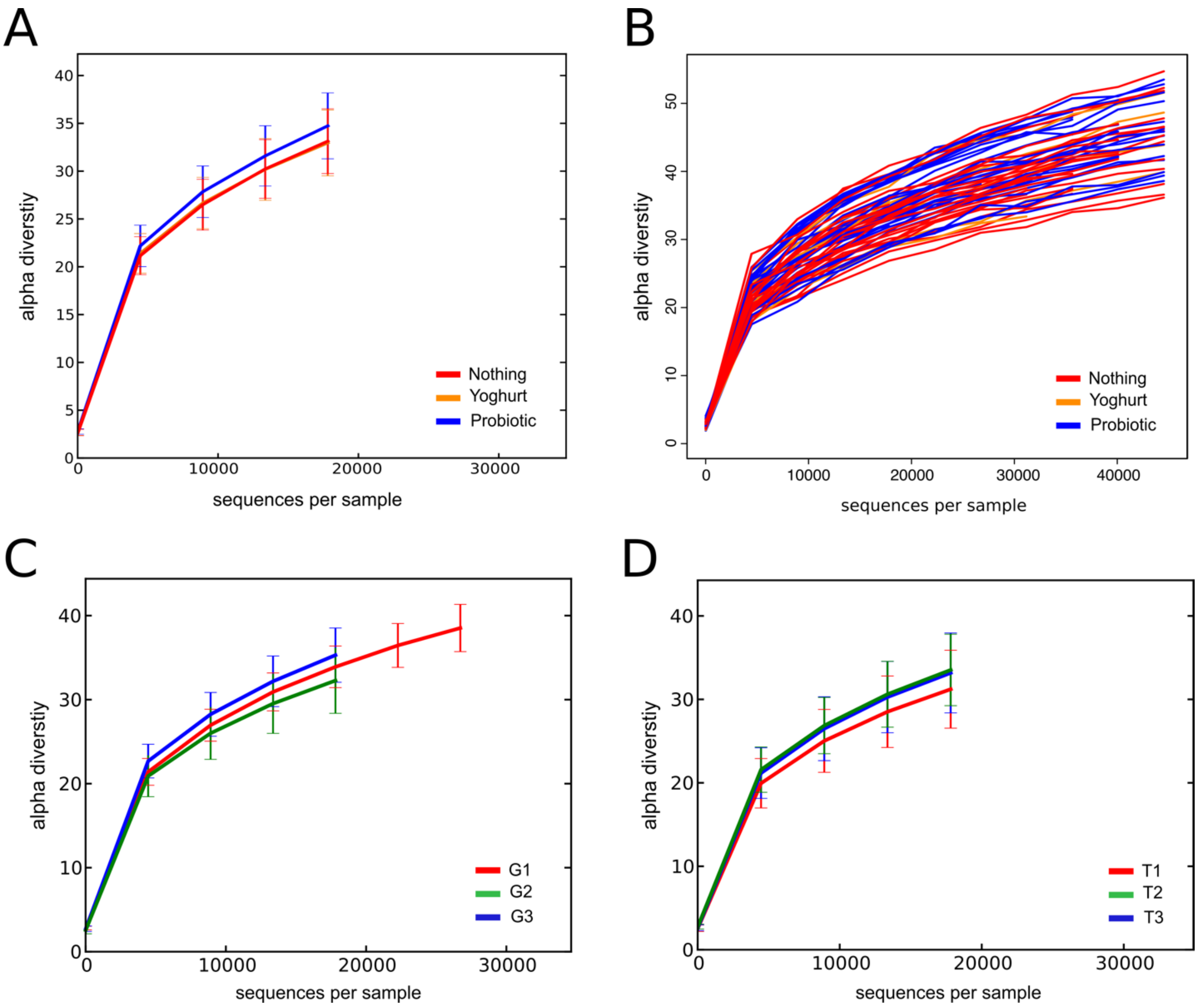

Supplementary Figure 2. Alpha rarefaction curves for the PD-Whole Tree metric, with samples colored by intake (**A** and **B**), by group (**C**), and timepoint (**D**).

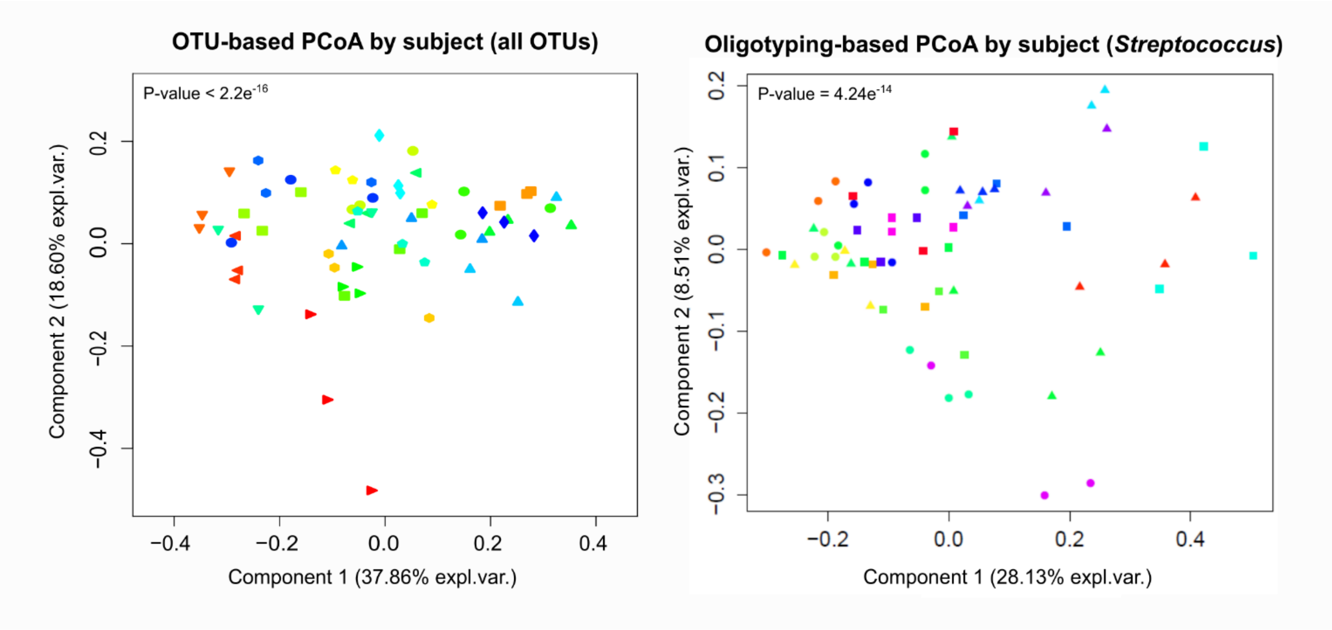

Supplementary Figure 3. OTU-based (including all OTUs, left) and oligotype-based PCoA by subject (including only *Streptococcus* oligotypes, right). OTU-based PCoA employs the weighted Unifrac metric while the oligotyping PCoA uses the Bray-Curtis metric.The proportion of explained variance for both principal components is indicated next to the component name. The indicated Wilcoxon test P-value represents the significance of the lower beta diversity observed within samples of the same subject with respect to samples of other subjects (intra subject vs inter subject beta diversity).

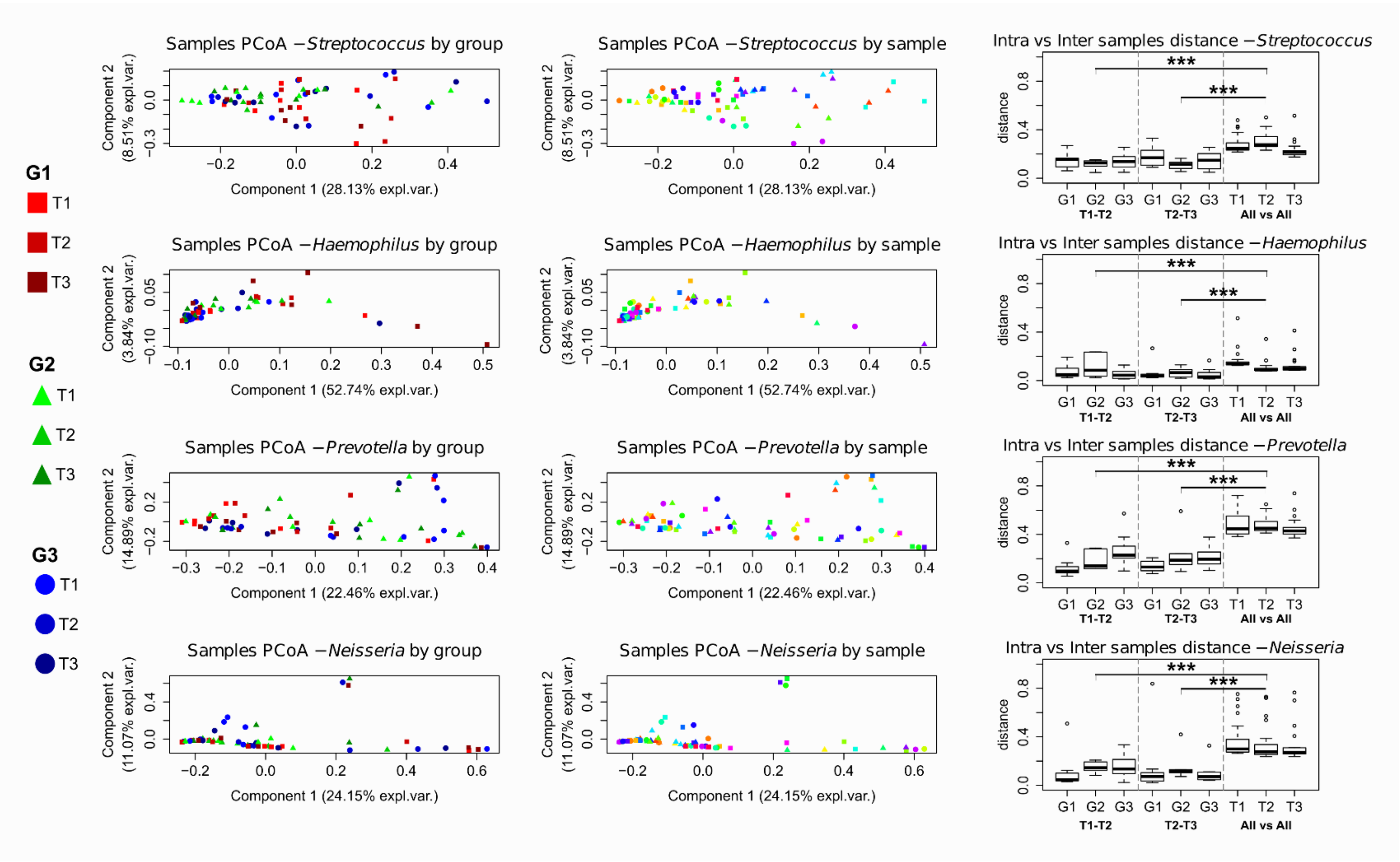

Supplementary Figure 4. Oligotype-based PCoA (Bray-Curtis metric) divided by group and sample (left and center columns), intra- and inter-samples distance comparison (right column) for the four main relevant genera (Streptococcus, Haemophilus, Prevotella and Neisseria). Intra-samples distance is defined as the distance between two time points of the same group, while the inter-samples distance is based on single timepoints across all groups. The proportion of explained variance for each principal component is indicated next to the component name. Distances diversity (Bray-Curtis metric) P-values are computed between intra-subject beta diversity and all diversity values (i.e. T1-T2 vs All, T2-T3 vs All) by using the Wilcoxon test (*** indicates P-value <.001).

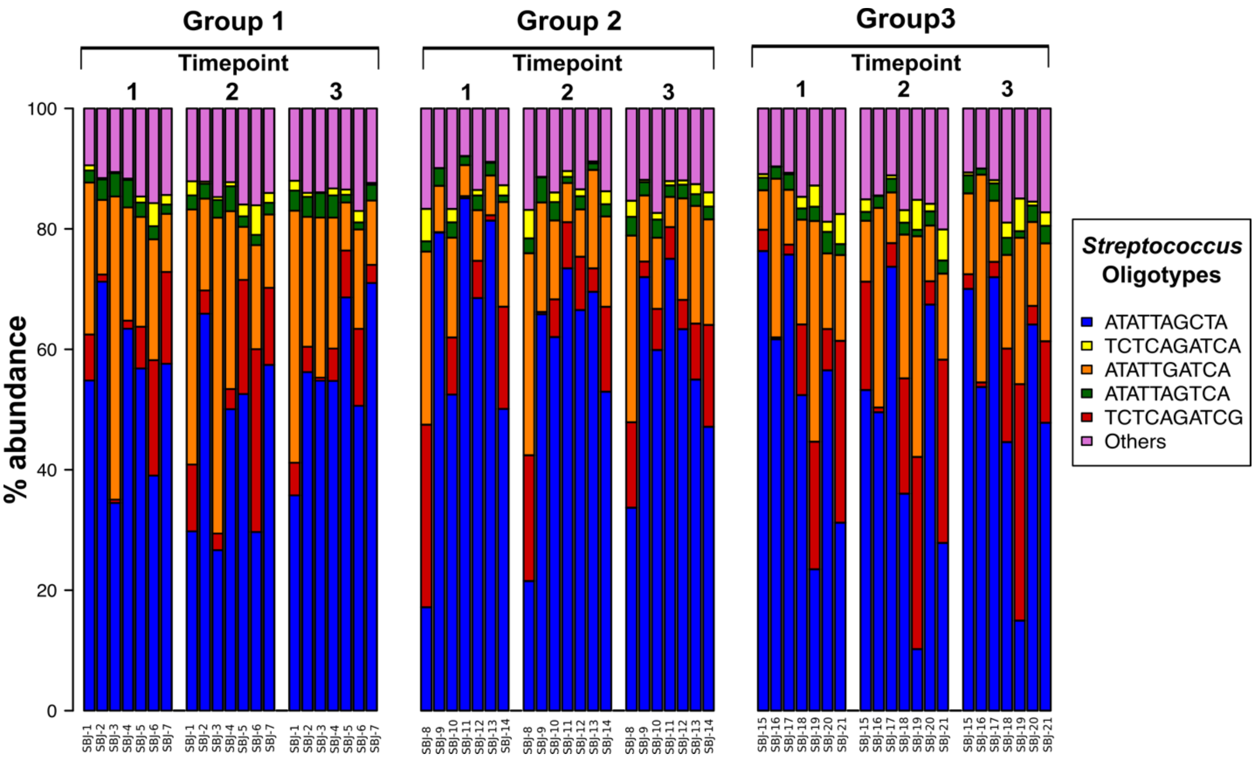

Supplementary Figure 5. Probiotic *Streptococcus* oligotypes across timepoints and study groups.

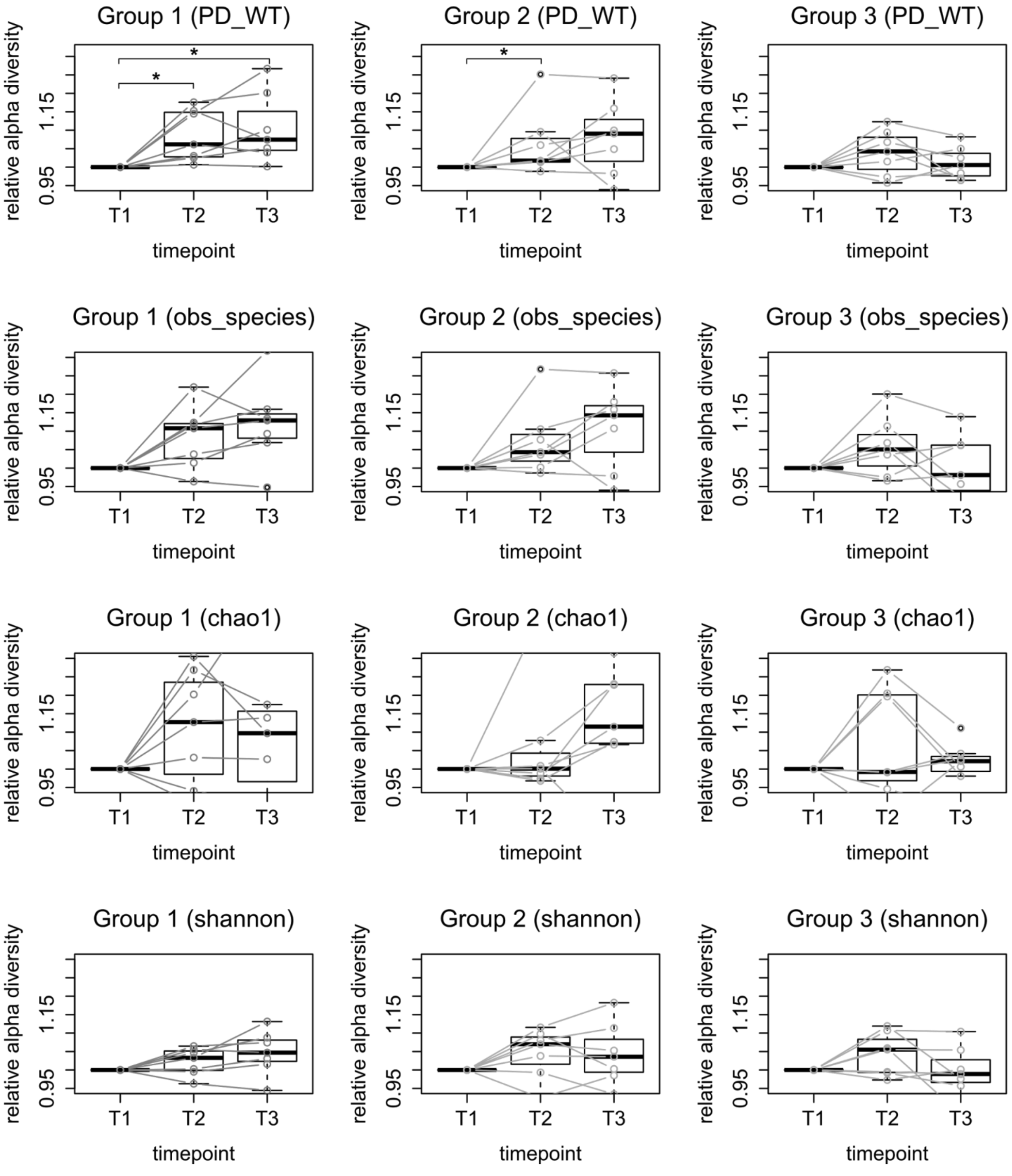
Group 1 (PD_WT)
Group 2 (PD_WT)
Group 3 (PD_WT)
Group 1 (obs_species)
Group 2 (obs_species)
Group 3 (obs_species)
Group 1 (chao1)
Group 2 (chao1)
Group 3 (chao1)
Group 1 (shannon)
Group 2 (shannon)
Group 3 (shannon)
relative alpha diversity
0.95
1.15
T1
T2
T3
timepoint
*

Supplementary Figure 6. Alpha diversity computed with four different metrics (PD_Whole Tree, Observed species, Chao1 and Shannon) on the three study groups (* for Wilcoxon test P-values <.05).

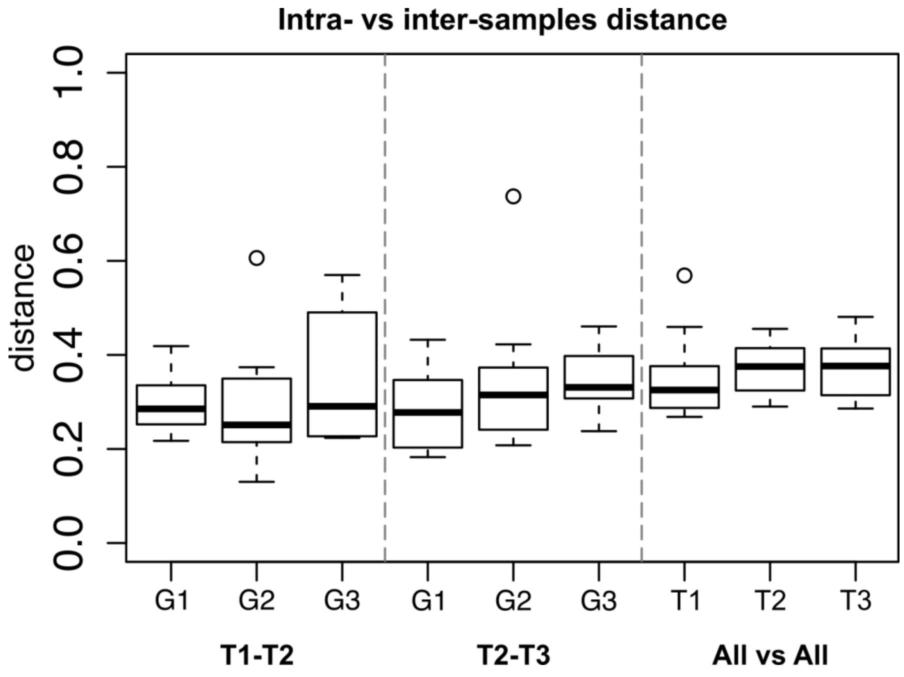

Supplementary Figure 7. OTU-based (beta diversity computed with the weighted Unifrac metric) inter-sample (difference between samples in each timepoint across groups) and intra-sample distances (variation of each group between timepoints). No statistically significant values were found.

Supplementary Tables

#SampleID	BarcodeSequence	Treatment	Timepoint	CombinedTreatmentTimepoint	Subject
S1	ACTTCCAACTTC	2	3	2_3	1
S2	TATACCGCTGCG	2	2	2_2	1
S3	GGCCAGTTCCTA	2	1	2_1	1
S4	GAGCCATCTGTA	1	3	1_3	2
S5	CAACTCCCGTGA	1	2	1_2	2
S6	CGAGCAATCCTA	1	1	1_1	2
S7	TAACGTGTGTGC	3	3	3_3	3
S8	TACAGCGCATAC	3	2	3_2	3
S9	CTGCTATTCCTC	3	1	3_1	4
S10	TGCGCTGAATGT	NA	1	NA_1	5
S11	ATGGGTTCCGTC	H2O	-	H2O_-	-
S12	ACGAGACTGATT	3	1	3_1	3
S13	CGTAATTGCCGC	3	1	3_1	6
S14	AGGCTTACGTGT	1	2	1_2	7
S15	TGTCGCAAATAG	1	1	1_1	7
S16	CATTCGTGGCGT	3	2	3_2	6
S17	TCGACATCTCTT	2	3	2_3	8
S18	AGTTACGAGCTA	H2O	-	H2O_-	-
S19	GCAACACCATCC	2	3	2_3	9
S20	GAATACCAAGTC	2	2	2_2	9
S21	GATCCCACGTAC	YOGURT	-	YOGURT_-	-
S22	GGACTTCCAGCT	1	3	1_3	7
S23	GTGGTGGTTTCC	3	3	3_3	6
S24	AGCGGAGGTTAG	2	1	2_1	9
S25	GATTCCGGCTCA	1	3	1_3	10
S26	GCTCGAAGATTC	1	2	1_2	10
S27	GTCGACAGAGGA	1	1	1_1	10
S28	TGTGAATTCGGA	NA	3	NA_3	11
S29	ACCATAGCTCCG	NA	2	NA_2	11
S30	AGATTGACCAAC	NA	1	NA_1	11
S31	CAAACAACAGCT	H2O	-	H2O_-	-
S32	ACCAGTGACTCA	NA	3	NA_3	12
S33	TTGGCTCTATTC	3	3	3_3	13
S34	GTTGTTCTGGGA	3	2	3_2	13

S35	GTACGATATGAC	3	1	3_1	13
S36	GTCGTGTAGCCT	NA	2	NA_2	12
S37	GTGTTGTCGTGC	3	1R	3_1R	14
S38	ACAATAGACACC	3	2	3_2	14
S39	CTATCTCCTGTC	3	3	3_3	14
S40	AAGGCGCTCCTT	-	-	-_-	-
S41	TACGAGCCCTAA	-	-	-_-	-
S42	GTATCTGCGCGT	-	-	-_-	-
S43	CCAATACGCCTG	-	-	-_-	-
S44	AATTGTGTCGGA	-	-	-_-	-
S45	TGTAACGCCGAT	H2O	-	H2O_-	-
S46	GTTCTCTTCTCG	H2O	-	H2O_-	-
S47	AACTAGTTCAGG	3	1	3_1	14
S48	ATCACCAGGTGT	-	-	-_-	-
S49	TCTCTACCACTC	2	1R	2_1R	15
S50	CATCCCTCTACT	2	1	2_1	15
S51	TACTACGTGGCC	H2O	-	H2O_-	-
S52	GAACACTTTGGA	1	3	1_3	16
S53	GCATATGCACTG	1	2	1_2	16
S54	GCGATATATCGC	1	1R	1_1R	16
S55	GTAGATCGTGTA	1	1	1_1	16
S56	ATCCTTTGGTTC	2	2	2_2	8
S57	CTCACAACCGTG	PROBIO	-	PROBIO_-	-
S58	TAGTATGCGCAA	2	3	2_3	15
S59	GGTGACTAGTTC	2	2	2_2	15
S60	TCCCTTGTCTCC	2	1	2_1	8
S61	CCACAGATCGAT	1	3	1_3	17
S62	CGGTCAATTGAC	1	2	1_2	17
S63	ACTCACAGGAAT	1	1	1_1	17
S64	TAATACGGATCG	1	3	1_3	18
S65	CACTACGCTAGA	1	2	1_2	18
S66	CGAGGGAAAGTC	1	1	1_1	18
S67	GATCTGCGATCC	2	3	2_3	19
S68	TGCATACACTGG	2	2	2_2	19
S69	AGCAGAACATCT	2	2	2_2	20
S70	CGTAAGATGCCT	2	1	2_1	20
S71	ATTCTGCCGAAG	H2O	-	H2O_-	-
S72	TGGTCAACGATA	2	1	2_1	19
S73	TATCGACACAAG	3	1	3_1	21

S74	GTGGAGTCTCAT	1	2	1_2	22
S75	ATGATGAGCCTC	1	1	1_1	22
S76	TCGGAATTAGAC	3	1	3_1	23
S77	TGCAGTCCTCGA	1	3	1_3	22
S78	CAAATTCGGGAT	H2O	-	H2O_-	-
S79	CAGCTCATCAGC	2	3	2_3	20
S80	AGTCGAACGAGG	3	3	3_3	23
S81	TGGAGTAGGTGG	NA	1	NA_1	12
S82	GCGTTCTAGCTG	3	3	3_3	21
S83	AGCATGTCCCGT	3	2	3_2	21
S84	ATCGCACAGTAA	3	2	3_2	23
S85	CTCACCTAGGAA	-	-	-_-	-
S86	AGTTGAGGCATT	NA	3	NA_3	24
S87	GATGTTCGCTAG	2	3	2_3	25
S88	TTGGGTACACGT	3	3	3_3	4
S89	TTGCGTTAGCAG	NA	2	NA_2	24
S90	AGTCGTGCACAT	2	2	2_2	25
S91	CATTATGGCGTG	3	2	3_2	4
S92	ACCGGTATGTAC	NA	1	NA_1	24
S93	ATGTCACCGCTG	-	-	-_-	-
S94	ATGGCTGTCAGT	-	-	-_-	-
S95	TAGGCATGCTTG	-	-	-_-	-
S96	GCTGTACGGATT	2	1	2_1	25

Supplementary Table 1. Complete list of sample-specific barcodes

Sample	Reads number
**S1**	50526
**S10**	46977
**S11**	6665
**S12**	51079
**S13**	20495
**S14**	27616
**S15**	54144
**S16**	52966
**S17**	48616
**S18**	5689

**S19**	27388
**S2**	35639
**S20**	45784
**S21**	11971
**S22**	57370
**S23**	38848
**S24**	48008
**S25**	44994
**S26**	45549
**S27**	37001
**S28**	49413
**S29**	42653
**S3**	46768
**S30**	53523
**S31**	8907
**S32**	43243
**S33**	50998
**S34**	45130
**S35**	44395
**S36**	36871
**S37**	25443
**S38**	45526
**S39**	18892
**S4**	43149
**S45**	5094
**S46**	9432
**S47**	38238
**S49**	28819
**S5**	44005
**S50**	53621
**S51**	3454
**S52**	49281
**S53**	53320
**S54**	39694
**S55**	54313
**S56**	46405
**S57**	56272
**S58**	46044
**S59**	54384

**S6**	42087
**S60**	54205
**S61**	45554
**S62**	51480
**S63**	36904
**S64**	33864
**S65**	44308
**S66**	32996
**S67**	54979
**S68**	19663
**S69**	54256
**S7**	40795
**S70**	45037
**S71**	1319
**S72**	55541
**S73**	36903
**S74**	50243
**S75**	49103
**S76**	46690
**S77**	43081
**S78**	5738
**S79**	49973
**S8**	39161
**S80**	48575
**S81**	24173
**S82**	38928
**S83**	42462
**S84**	40829
**S86**	35517
**S87**	45406
**S88**	50658
**S89**	44453
**S9**	38436
**S90**	45953
**S91**	27628
**S92**	38070
**S96**	32260

106

Supplementary Table 2. Total million reads per sample
